# A High Esterifying Enzyme-Producing *Rhizopus* Strain for Fortified Daqu: Screening, Preparation, and Microbial Community Characterization

**DOI:** 10.3390/foods15071213

**Published:** 2026-04-02

**Authors:** Qihao Peng, Chunhui Wei, Jun Xie, Zhuolin Yi, Zhiqiang Ren

**Affiliations:** 1 Sichuan Provincial Key Laboratory of Brewing Science and Technology, Sichuan University of Science & Engineering, Yibin 644000, China; 13896002828@163.com (Q.P.);; 2 Agricultural Microbial Agents Key Laboratory of Sichuan Province, Chengdu Institute of Biology, Chinese Academy of Sciences, Chengdu 610213, China

**Keywords:** high-throughput sequencing, *Rhizopus oryzae*, pure-culture Fuqu, fortified Daqu, solid-state fermentation, analysis of the community composition

## Abstract

To explore microbial resources for ester production in sub-high-temperature Daqu, this study first established that the esterifying enzyme activity in Daqu predominantly originated from fungi, with *Rhizopus* being the dominant fungal genus. Six *Rhizopus* strains capable of decomposing esters were isolated and purified from Daqu. Following secondary screening, strain M1 exhibited the highest esterification activity (40.26 U/mL) and was identified as *Rhizopus oryzae* based on morphological characteristics and molecular biological analyses. This strain was subsequently designated as *Rhizopus oryzae* M1 (*R. oryzae* M1). Using mycelial powder of strain M1 as the inoculum and sterilized wheat bran as the substrate, a pure-culture Fuqu was prepared. Orthogonal array design experiments were conducted to optimize the preparation process of this Fuqu, using esterifying enzyme activity as the evaluation index. Under the optimal conditions, the spore count and esterification activity of the pure-culture Fuqu reached 1.73 × 10^9^ CFU/g and 80.13 U/g, respectively. This pure-culture Fuqu was subsequently used as an inoculum to produce fortified Daqu. Following orthogonal optimization of the Daqu preparation process, the esterification activity of the fortified Daqu reached 103.22 U/g, and its key physicochemical indices met the requirements for high-quality sub-high-temperature Daqu. Analysis of the microbial community structure revealed that *Rhizopus* was the dominant fungal genus in the fortified Daqu, with its relative abundance increased by 35% compared to the non-fortified Daqu. Consistent with this, the esterifying enzyme activity of the fortified Daqu was 51.79% higher, suggesting that *Rhizopus* may have been largely responsible for the increase in esterification capacity. In laboratory-scale Baijiu brewing trials, this fortified Daqu produced a base Baijiu with a total ester content of 2.74 g/L, representing a 40.5% increase over the non-fortified Daqu and further confirming the pivotal role of *Rhizopus* in driving the esterifying enzyme activity. This study successfully screened a high esterifying enzyme-producing strain, *R. oryzae* M1, systematically optimized its enzyme production and Qu-making processes, and provides an excellent microbial strain and process reference for the preparation of fortified Daqu and the enhancement of Baijiu flavor.

## 1. Introduction

Baijiu, a traditional Chinese distilled spirit, is produced from cereal grains using saccharification and fermentation agents such as Daqu, Xiaoqu, Fuqu, or enzyme preparations combined with yeasts [1]. Daqu, the core starter for major Baijiu styles, is manufactured by pressing crushed wheat, barley, and/or peas into bricks, which are then subjected to natural enrichment of microorganisms and enzymes under controlled temperature and humidity [2]. The resulting enzyme system, including amylases, saccharifying enzymes, proteases and so on, is utilized to degrade macromolecules in the raw materials into fermentable sugars and amino acids, which are subsequently converted by yeasts and other microbes into ethanol and a diverse array of flavor compounds. Among various Daqu types, sub-high-temperature Daqu (with a peak curing temperature of 50–60 °C) serves as the essential starter for strong-aroma Baijiu and is characterized by its rich aroma and balanced saccharification and fermentation capabilities. Its microbial community is dominated by thermotolerant fungi such as *Thermoascus aurantiacus*, *Dipodascus geotrichum*, *Wickerhamomyces anomalus*, and *Rhizopus* spp., along with bacteria including *Acinetobacter johnsonii*, *Lactobacillus sakei*, and *Bacillus* spp., all of which are closely linked to flavor compound formation [3,4]. The quality of sub-high-temperature Daqu is recognized to play a crucial role in improving liquor yield and the proportion of premium liquor [5]. Despite the critical importance of Daqu, its traditional production through natural fermentation is associated with a complex and unstable microbial community structure. This inherent variability is considered to lead to significant fluctuations in key enzyme activities, particularly esterifying enzyme activity, which is directly associated with flavor compound formation. Such instability is widely regarded as a major technical bottleneck that severely limits the consistency and overall improvement of base Baijiu quality [6]. Consequently, how to directionally enhance and stabilize the esterification capacity of Daqu while ensuring that saccharifying and liquefying activities meet required levels has emerged as a critical research topic of significant industry concern. In recent years, the preparation of fortified Daqu through the exogenous addition of functional microorganisms has been recognized as a promising strategy for regulating Daqu quality [7].

The present study focuses on the development and utilization of ester-producing microbial resources in sub-high-temperature Daqu. To date, research on ester-producing molds in Daqu has primarily been concentrated on the genus *Monascus*, with considerable attention being given to their ester-producing characteristics and applications. In contrast, systematic investigations of *Rhizopus* species, which are considered to possess comparable ester-producing potential, remain relatively limited, and their specific mechanisms of action in Baijiu flavor formation and process adaptability have not yet been fully elucidated. In response to these practical challenges, this study isolated microorganisms with high esterifying enzyme activity from sub-high-temperature Daqu and applied them in Baijiu fermentation. First, a strain with high esterifying enzyme activity was screened and identified from sub-high-temperature Daqu. Subsequently, pure-culture Fuqu was prepared using this strain as the inoculum, and the preparation process was optimized. Finally, the optimized Fuqu was utilized to produce fortified Daqu, and its key performance indicators, particularly esterifying enzyme activity, were systematically evaluated. Solid-state fermentation experiments were then conducted to validate its effectiveness in enhancing the total ester content of the base Baijiu. This study is aimed at providing a theoretical basis and microbial strain support for the diversified development of microbial resources in sub-high-temperature Daqu, while offering technical references for the directed application of *Rhizopus* in Baijiu fermentation. The findings are expected to contribute to the advancement of fermentation microbiology theory and the development of microbial regulation technologies for traditional fermented foods.

## 2. Materials and Methods

### 2.1. Materials and Instruments

Sub-high-temperature Daqu was obtained from a distillery in Yibin, Sichuan Province, China. Japonica sorghum, rice husks, wheat, and wheat bran were purchased from local commercial sources. Potato dextrose agar (PDA) medium was procured from Chengdu Kelong Chemical Reagent Factory. Tributyrin was obtained from Shanghai Macklin Biochemical Technology Co., Ltd. (Shanghai, China) Lactophenol cotton blue stain was purchased from Fuzhou Feijing Biotechnology Co., Ltd. (Fuzhou, China). All other chemicals and reagents used were of analytical grade.

PDA medium was prepared with the following components per liter: potato infusion powder, 6.0 g, glucose, 20.0 g, and agar, 20.0 g, made up to 1.0 L with distilled water. After sterilization, chloramphenicol was added to a final concentration of 25 μg/mL. Tributyrin medium was composed of 0.4% (*v*/*v*) tributyrin, with the remaining components identical to those of the PDA medium. LB medium was prepared with the following components per liter: tryptone 10 g, NaCl 10 g, yeast extract 5 g, and agar 20 g (for solid medium), made up to 1.0 L with distilled water. After sterilization, cycloheximide was added to a final concentration of 50 μg/mL.

Electrophoresis apparatus (Model 164-5070, Bio-Rad Laboratories, Hercules, CA, USA), PCR thermal cycler (Bio-Rad Laboratories), and ChemiDoc XPS+ gel imaging system (Bio-Rad Laboratories) were used for nucleic acid analysis. Microscopic observation and image acquisition were performed using a Leica microscope (Leica Microsystems, Wetzlar, Germany). Gas chromatographic analysis was conducted using an Agilent 8890 GC system (Agilent Technologies, Santa Clara, CA, USA) equipped with a flame ionization detector (FID).

### 2.2. Identification of the Primary Contributor to Esterifying Enzyme Activity in Sub-High Temperature Daqu

To investigate the primary source of esterifying enzyme activity in sub-high-temperature Daqu, the enzyme activities derived from bacteria and fungi were determined separately. Daqu samples were crushed and passed through a 40-mesh sieve. Separate 1.0 g aliquots of the sample were inoculated into 100 mL of sterilized LB liquid medium (incubated at 37 °C with shaking at 180 rpm for 48 h) and PDB (Potato Dextrose Broth) medium (incubated at 30 °C with shaking at 180 rpm for 48 h). Following incubation, the fermentation broths were centrifuged at 8000× *g* for 15 min at 4 °C and filtered through a 0.22 μm membrane filter, after which the esterifying enzyme activity was determined.

DNA extraction was performed following the in situ direct extraction method described by Wu et al. [8]. Qualified DNA samples were sent to Shanghai Majorbio Bio-Pharm Technology Co., Ltd. (Shanghai, China) for amplification of the fungal ITS region and subsequent sequencing on the PacBio Sequel II system. Data analysis was conducted using the DADA2 plugin within the Qiime2 v2020.2 for amplicon sequence variant (ASV) clustering. Species annotation was performed based on the UNITE database.

### 2.3. Screening and Identification of Rhizopus Strains

A 10.0 g aliquot of Daqu powder was weighed and added to 90 mL of sterile water. The mixture was shaken at 150 rpm for 30 min at 30 °C to prepare a microbial suspension. The suspension was subjected to 10-fold serial dilutions, and 100 µL aliquots from appropriate dilutions were spread onto PDA plates. The plates were incubated inverted at 30 °C for 48 h. Single colonies were selected based on morphological differences and purified by streaking on PDA plates; this purification process was repeated three times. The purified mold isolates were inoculated onto PDA slants, incubated at 30 °C for 48 h, and then stored at 4 °C for subsequent experiments.

Primary screening: Primary screening of ester-producing microorganisms was conducted using the tributyrin plate assay, following a modified method described by Farooq et al. [9]. The ratio of the clear zone diameter to the colony diameter (D/d) was used as a preliminary indicator to assess the hydrolytic activity of the isolates.

Secondary screening: A small amount of mycelium was inoculated into sterilized PDB medium and cultured at 30 °C with shaking at 180 rpm for 48 h. Subsequently, the esterifying enzyme activity was determined.

Morphological identification: The strain was inoculated onto PDA medium and incubated at 30 °C for 48 h. A small amount of mycelium and spores from the purified strain were then picked using a sterile inoculation needle, placed onto a glass slide stained with lactophenol cotton blue, gently dispersed with the needle, and covered with a coverslip. The prepared slide was examined under an optical microscope, and microscopic morphological characteristics were observed and recorded using a 40× objective lens [10].

Molecular biological identification: The identification was performed following the method described by Xu et al. [11] with slight modifications. The strain was inoculated into sterilized PDB medium and cultured at 30 °C with shaking at 180 rpm for 48 h. Following incubation, the mycelial pellets were collected by filtration through sterile filter paper. Two to three mycelial pellets with uniform growth status and approximately 3–5 mm in diameter were selected and placed into a 2 mL centrifuge tube. PBS buffer (phosphate-buffered saline 1.5 mL) was added, and the mixture was vortexed for 10 s, followed by centrifugation at 12,000× *g* for 5 min. The supernatant was discarded. This washing procedure was repeated three times, and the mycelial pellet precipitate was collected for subsequent DNA extraction. Genomic amplification was performed in a 50 μL reaction system using NS1 and NS4 as primers. The reaction mixture contained 1 μL of NS1, 1 μL of NS4, 1 μL of template DNA, 25 μL of Taq PCR Master Mix, and 22 μL of ultrapure water.

The resulting PCR products were sent to Sangon Biotech for sequencing. Following sequence editing, homology alignment was conducted using BLAST v2.17.0 against the NCBI database, and sequences from highly homologous related strains were selected as references. A phylogenetic tree was constructed using MEGA v11 software with the neighbor-joining method, and branch node support was evaluated using the bootstrap method (1000 replicates).

### 2.4. Preparation and Process Optimization of Pure-Culture Fuqu Using R. oryzae M1

Preparation of mycelial powder. A small amount of *R. oryzae* M1 mycelium was inoculated into sterilized PDB medium and cultured at 30 °C with shaking at 180 rpm for 48 h. After incubation, the mycelial pellets were collected by filtration, dried at 40 °C, and ground into powder.

Pure-culture Fuqu preparation. Wheat bran (the wheat bran used in this study had a moisture content of approximately 12%) and distilled water were mixed at a material-to-water ratio of 1:0.9 (*w*/*v*, bran weight: water), sterilized at 121 °C for 20 min, and cooled to room temperature. The mycelial powder was then inoculated at predetermined amounts (0.4%, based on the weight of the hydrated wheat bran), mixed thoroughly, and incubated at 30 °C for 72 h, with a turning operation performed at 36 h.

Single-factor experiments were conducted to investigate the effects of inoculation amount (0.1, 0.2, 0.3, 0.4, 0.5%), incubation temperature (24, 27, 30, 33, 36 °C), and Huangshui addition amount (0, 5, 10, 15, 20%) on esterifying enzyme activity (primary indicator), saccharifying enzyme activity, and liquefying enzyme activity. Huangshui is a brownish, viscous liquid byproduct that percolates from fermented grains and accumulates at the bottom of the pit during strong-aroma Baijiu fermentation, rich in organic acids, residual starch, and functional microorganisms. Pre-experiments indicated that inoculation below 0.1% resulted in retarded mycelial growth and a significantly prolonged enzyme production cycle, whereas inoculation above 0.5% led to excessive mycelial proliferation, reduced substrate aeration, and, consequently, decreased the esterifying enzyme activity of the Fuqu. Each treatment was performed in triplicate. When investigating the effect of one factor, the other two were fixed as follows: for inoculation amount, the temperature was fixed at 30 °C and Huangshui addition at 0%; for incubation temperature, the inoculation amount was fixed at 0.4% and Huangshui addition at 0%; and for Huangshui addition amount, the inoculation amount was fixed at 0.4% and incubation temperature at 30 °C. Based on the results of the single-factor experiments, a three-factor, three-level orthogonal experiment was designed according to the L_9_(3^3^) orthogonal array, with the Huangshui addition amount (A), inoculation amount (B), and incubation temperature (C) as the influencing factors and the esterifying enzyme activity as the evaluation index.

### 2.5. Preparation and Process Optimization of Fortified Daqu Using R. oryzae M1

Preparation of fortified Daqu: Wheat grains free from mold and with plump kernels were selected, soaked for 12 h, drained of surface moisture, and then crushed using a grinder. The resulting material was passed through a 40-mesh standard sieve, and the sieved fraction was collected as the Daqu-making substrate. Subsequently, pure water was added to the substrate at a water-to-substrate mass ratio of 0.4:1, and the mixture was uniformly mixed to achieve the target moisture content. Starter Daqu powder and pure-culture Fuqu powder was then sequentially added at a predetermined inoculation amount (3%, based on the total weight of the hydrated and mixed wheat) and thoroughly mixed to form the Daqu mixture. The mixture was filled into standard molds and pressed into shape. After demolding, Daqu bricks of uniform specifications were obtained. The Daqu bricks were transferred to a constant temperature and humidity incubation room and subjected to temperature-controlled fermentation for 28 d under set temperature and humidity conditions. The control group was prepared following the same procedure, except that an equal mass of sterilized dry wheat bran was used in place of the pure-culture Fuqu powder.

Single-factor experiments were conducted to investigate the effects of pure-culture Fuqu inoculation amount (1, 2, 3, 4, 5%), starter Daqu addition amount (1, 2, 3, 4, 5%), and Huangshui addition amount (0, 2, 4, 6, 8%) on esterifying enzyme activity, saccharifying enzyme activity, and liquefying enzyme activity. Pure-culture Fuqu was used as the seed inoculum, with the inoculation amount set within the range of 1–5%. Given the larger particle size, lower water activity, and the presence of a complex indigenous microbial community in Daqu raw materials, a higher initial inoculum was considered necessary to ensure effective colonization by the target functional strain; however, inoculation exceeding 5% conversely led to a decrease in the esterifying enzyme activity of the Daqu. All treatments were performed in triplicate. When the effect of one factor was investigated, the other two factors were fixed as follows: for inoculation amount, starter Daqu addition amount was fixed at 3% and Huangshui addition amount at 0%; for starter Daqu addition amount, inoculation amount was fixed at 3% and Huangshui addition amount at 0%; for Huangshui addition amount, inoculation amount was fixed at 3% and starter Daqu addition amount at 3%. Based on the results of the single-factor experiments, a three-factor, three-level orthogonal experiment was designed according to the L_9_(3^3^) orthogonal array, with Huangshui addition amount (A), inoculation amount (B), and starter Daqu addition amount (C) as the influencing factors, and esterifying enzyme activity as the evaluation index.

### 2.6. Laboratory-Scale Baijiu Brewing

Solid-state fermentation: Japonica sorghum grains with plump kernels and free from mold were selected, cleaned to remove impurities, crushed, and passed through a 20-mesh sieve. Huangshui (obtained from a strong-aroma liquor distillery in Yibin) was added at 60% of the sorghum weight, and the mixture was soaked for 2 h. Rice husks were then added at 20% of the sorghum weight. After thorough mixing, the mixture was transferred to a steaming vessel and steamed for 60 min. Boiling water was immediately added to the fermented grains to adjust the moisture content to 60%. Following cooling and spreading, Daqu was added at 10% of the fermented grain weight, mixed thoroughly, and fermented at a constant temperature of 32 °C for 45 d. The fermented grains were subjected to distillation using a conventional solid-state Baijiu process [12].

### 2.7. Analytical Methods

Determination of esterifying enzyme activity of the strain: After liquid culture of the strain for a specified period, the liquid culture medium was filtered to obtain the fermentation broth for subsequent analysis. Cyclohexane (4.0 mL), ethanol (1 mL), and acetic acid (0.3 mL) were added to a sealed test tube, followed by the addition of 200 μL of crude enzyme extract. The esterification reaction was conducted at 33 °C for 96 h. After the reaction, 0.5 mL of the supernatant was transferred to a clean beaker, and 5 mL of deionized water and two drops of phenolphthalein indicator were added. The mixture was titrated with 0.10 mol/L NaOH standard solution until a faint pink color persisted for 30 s. The consumption of acetic acid was calculated based on the volume of NaOH consumed.

Definition of esterifying enzyme activity unit for the strain fermentation broth: One unit of enzyme activity (U/mL) was defined as the amount of enzyme required to synthesize 1 μmol of ethyl acetate per hour in the esterification mixture. Determination of esterifying enzyme activity in Fuqu/Daqu: The esterification liquid was obtained using a solid-state esterification method, and the esterifying enzyme activity was subsequently determined by gas chromatography [13].

Definition of esterifying enzyme activity unit: One unit of enzyme activity (U/g) was defined as the amount of enzyme required to catalyze the synthesis of 1 μmol of ethyl acetate per hour per gram of pure-culture Fuqu or fortified Daqu. Gas chromatography conditions: An LZP-930 capillary chromatographic column (30 m × 0.32 mm × 10 μm) was used. The temperature program was set as follows: initial hold at 50 °C for 6 min, followed by an increase to 170 °C at a rate of 5 °C/min, with a final hold at 170 °C for 5 min. The injector temperature was 230 °C, and the injection volume was 1 μL in splitless mode.

Solid-state esterification: Cooked rice husks (1000 g), Huangshui (500 mL), anhydrous ethanol (500 mL), and pure-culture Fuqu/fortified Daqu (100 g) were added to an insulation bucket, mixed thoroughly, and fermented under constant temperature and humidity conditions at 30 °C for 14 d.

Esterification liquid: The fermented substrate was distilled, and 2000 mL of the initial distillate was collected. This initial distillate was then redistilled to obtain 500 mL of esterification liquid. An aliquot of 1.0 mL was accurately measured as the sample for analysis.

Determination of spore count in Fuqu [14]: A 1.0 g aliquot of dried Fuqu sample was accurately weighed and placed into a conical flask, followed by the addition of 50 mL of sterile water. The mixture was shaken at 180 rpm for 30 min on a shaker to ensure adequate spore dispersion. Subsequently, the dispersed suspension was sampled, and spore counting was performed using a hemocytometer.

Determination of moisture content in Fuqu: A 5.0 g aliquot of the Fuqu sample to be tested was accurately weighed into a Petri dish and dried to constant weight in an oven set at 40 °C. The Petri dish was then covered, transferred to a desiccator, and cooled for 30 min before being weighed again. The moisture content was calculated according to the method described in reference [15].

Determination of saccharifying enzyme activity, liquefying enzyme activity, and fermentation power: The analyses were performed following the methods described by Song et al. [16]. The saccharifying enzyme activity was determined using the DNS colorimetric method; the liquefying enzyme activity was determined using the iodine colorimetric method; and the fermentation power was determined using the CO_2_ weight loss method.

Determination methods for total esters, total acids, and alcohol content: The analyses were performed according to the national standard method GB/T 10345—2022 [17] “Method of Analysis for Baijiu”. The total ester content was determined by the indicator method, and the total acid content was determined by the acid-base titration method. The alcohol content was determined by the alcohol meter method.

Liquor yield [18]: The output of the base Baijiu was determined by the weighing method. The liquor yield was calculated as the ratio of the output to the grain input.

### 2.8. Data Analysis

All experiments were performed in three independent biological replicates, with each biological replicate consisting of three technical replicates. Results were expressed as the mean ± standard deviation (SD). Data visualization was conducted using Origin 2022. Phylogenetic trees were constructed using MEGA 11 software. All statistical analyses were performed using R software. For comparisons between two groups, Student’s *t*-test was applied using the t.test() function. For comparisons involving more than two groups, one-way ANOVA was conducted with the aov() function, followed by Tukey’s HSD post hoc test using the TukeyHSD() function. A *p*-value < 0.05 was considered statistically significant.

## 3. Results and Discussion

### 3.1. Rhizopus Was Identified as the Primary Source of Esterifying Enzyme Activity in Sub-High-Temperature Daqu

**Figure 1:**
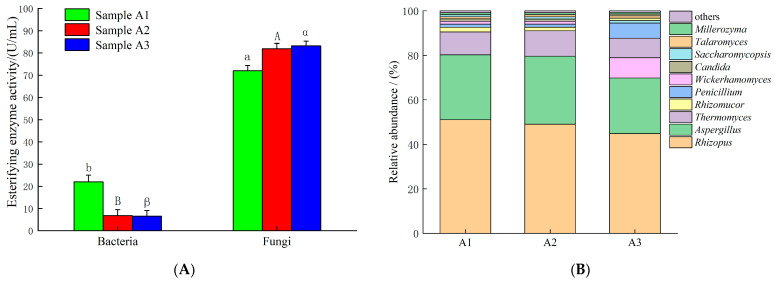
Fungal community structure in Daqu and the primary source of its esterifying enzyme activity. (**A**) Primary source of esterifying enzyme activity in Daqu; (**B**) analysis of the fungal community structure in Daqu. Values are presented as mean ± SD (*n* = 3). Different letters in the same column indicate significant differences at *p* < 0.05.

Microorganisms in Daqu were initially divided into bacterial and fungal groups using selective media, and the esterifying enzyme activity in the fermentation broth of each group was subsequently measured. As shown in Figure 1A, the esterifying enzyme activities in the fungal group were 71.4 ± 1.1 U/mL, 69.5 ± 1.2 U/mL, and 69.8 ± 1.0 U/mL, respectively, whereas those in the bacterial group were 23.4 ± 1.4 U/mL, 13.1 ± 1.1 U/mL, and 13.2 ± 1.0 U/mL. The esterifying enzyme activities in the fungal group were substantially higher than those in the bacterial group, and a significant difference between the two groups was confirmed by statistical analysis. These results indicate that fungi are likely the primary source of esterifying enzyme activity in sub-high-temperature Daqu.

The fungal community structure in Daqu was analyzed using high-throughput sequencing technology. As shown in Figure 1B, the dominant fungal genera in the three types of sub-high-temperature Daqu were *Rhizopus*, *Aspergillus*, and *Thermomyces*, among others. Among these, *Rhizopus* exhibited the highest relative abundance, accounting for 52.4%, 50.2%, and 44.1%, respectively. Given that the esterifying enzyme activity in Daqu was primarily derived from fungi, and *Rhizopus* was the dominant genus within the fungal community, the *Rhizopus* strains present in Daqu were considered likely to possess high esterifying enzyme activity [19]. Therefore, sample A1, which exhibited the highest relative abundance of *Rhizopus*, was selected for subsequent screening of *Rhizopus* strains.

### 3.2. Screening and Identification of the High Esterifying Enzyme-Producing Strain M1

**Figure 2:**
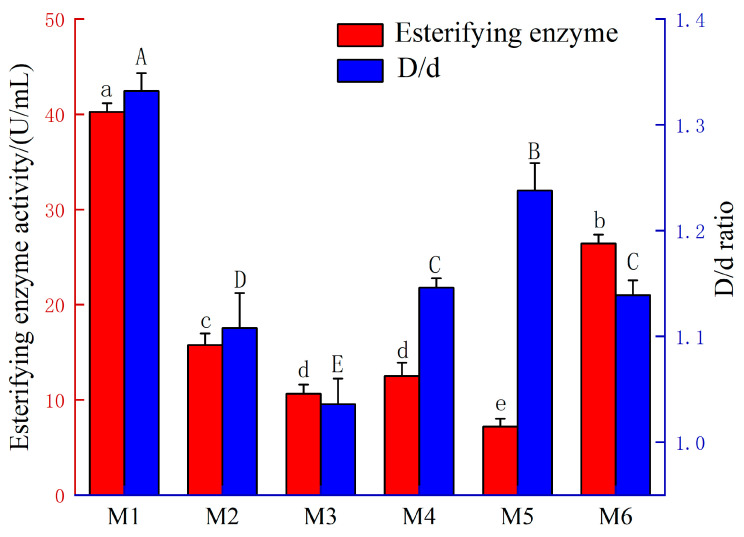
Esterifying enzyme activity and the ratio of transparent zone diameter to colony diameter (D/d) of six *Rhizopus* strains. Different letters in the same column indicate significant differences at *p* < 0.05.

In this study, the tributyrin plate assay [20] was used as a preliminary screening method to evaluate the hydrolytic potential of six *Rhizopus* strains isolated from Daqu, with the ratio of the transparent zone diameter to the colony diameter (D/d) employed as an indicator. Each strain was cultured in PDB medium under shaking conditions, and the esterifying enzyme activity in the fermentation broth was measured to assess the actual ester synthesis capacity. The results are presented in Figure 2, where transparent zones were observed for all six *Rhizopus* strains on tributyrin agar plates, with significant variations in D/d ratios among them. The highest D/d ratio of 1.332 ± 0.017 was exhibited by strain M1, indicating its strong hydrolytic potential. Significant differences in esterifying enzyme activity were similarly revealed by the liquid fermentation results; the highest activity of 40.26 U/mL was displayed by strain M1, which was significantly higher than that of the other strains, indicating its excellent ester synthesis capacity. Although relatively strong hydrolytic potential (high D/d ratio) was exhibited by strain M5, its esterifying enzyme activity was only 8.32 U/mL, reflecting limited ester synthesis capacity. This example demonstrates that hydrolytic activity is not representative of ester synthesis capacity and that the size of the transparent zone alone is insufficient for the accurate evaluation of the ester-producing potential of a strain. The determination of hydrolytic capacity is therefore suitable only for preliminary screening, whereas a comprehensive assessment of a strain’s ester-producing potential should be based on esterifying enzyme activity as the key indicator [21]. Based on its highest esterifying enzyme activity—the key indicator of ester-producing potential—strain M1 was therefore selected as the target strain for subsequent experiments.

**Figure 3:**
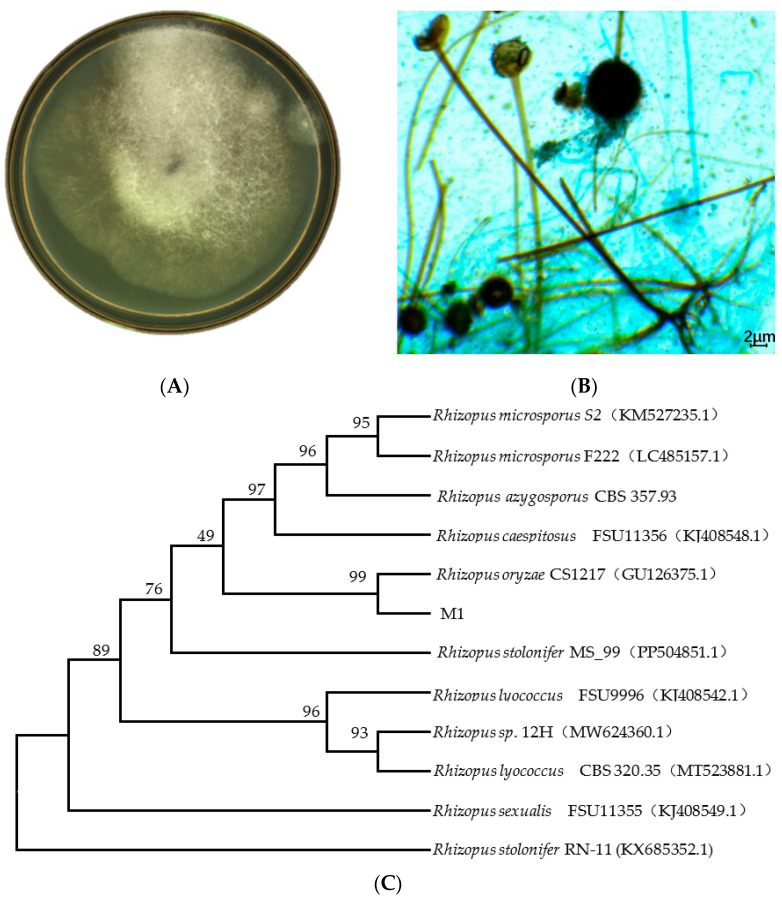
Identification of strain M1. (**A**) Colony morphology of strain M1. (**B**) Mycelial morphology of strain M1. (**C**) Phylogenetic tree of strain M1 with similar *Rhizopus* strains based on 18S rRNA gene sequences.

Colony morphology of strain M1 is shown in Figure 3A. After cultivation on PDA medium at 30 °C for 36 h, the colonies of strain M1 appeared circular and expanded rapidly. The colonies were highly fluffy, exhibiting a typical cotton-like morphology with a soft texture. Mycelial morphology of strain M1 is shown in Figure 3B. Strain M1 exhibited well-developed mycelia, characterized by typical coenocytic (non-septate) and multinucleate hyphae. Distinct stolons were observed, along with rhizoids and sporangiophores arising from the nodes. The sporangiophores were erect, non-septate, and terminated with spherical sporangia, which turned black upon maturation. These morphological characteristics were consistent with the typical description of the genus *Rhizopus*. Therefore, strain M1 was preliminarily identified as belonging to *Rhizopus* [22]. A phylogenetic tree of strain M1 was constructed based on the ITS gene sequence, and the results are shown in Figure 3C. As shown in Figure 3C, strain M1 shared 99% homology with *Rhizopus oryzae* and clustered in the same branch, indicating the closest phylogenetic relationship. Therefore, strain M1 was identified as *Rhizopus oryzae*. *Rhizopus oryzae* is a filamentous fungus belonging to the family Mucoraceae. In traditional classification, it is placed within the zygomycetous fungi, whereas modern taxonomy has assigned it to the phylum Mucoromycota [23].

### 3.3. Optimization of Preparation Conditions for R. oryzae M1 Pure-Culture Fuqu

**Figure 4:**
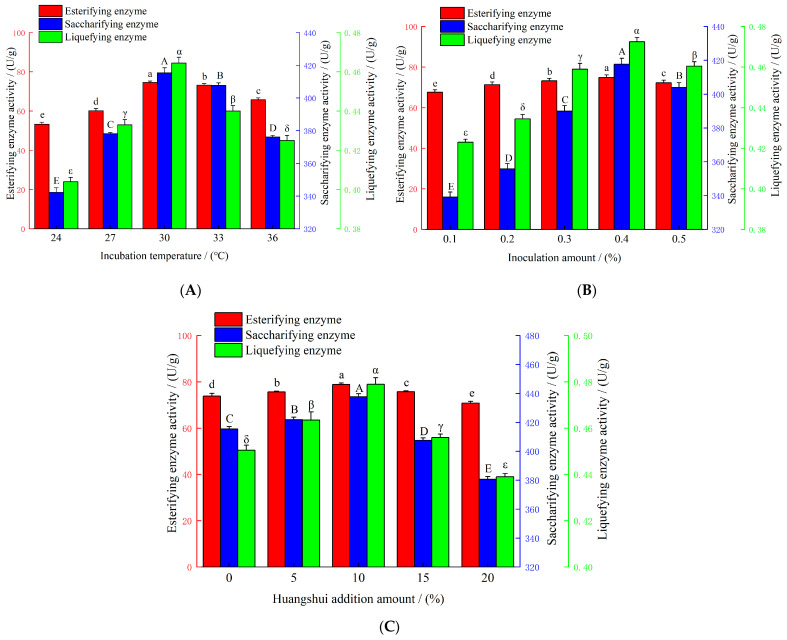
Effects of different factors on enzyme activities of *R. oryzae* M1 pure-culture Fuqu. (**A**) Effect of incubation temperature on the esterifying enzyme activity, saccharifying enzyme activity, and liquefying enzyme activity of *R*. oryzae M1 Pure-culture Fuqu. (**B**) Effect of inoculation amount on the esterifying, saccharifying, and liquefying enzyme activities of *R*. oryzae M1 pure-culture Fuqu. (**C**) Effect of Huangshui addition amount on the esterifying, saccharifying, and liquefying enzyme activities of *R. oryzae* M1 pure-culture Fuqu. Different letters indicate significant differences at *p* < 0.05. The inoculation amount was based on mycelial powder of *R. oryzae* M1.

The effects of incubation temperature on the enzyme activities of *R. oryzae* M1 pure-culture Fuqu are shown in Figure 4A. As the incubation temperature increased, the esterifying enzyme activity (key evaluation index, hereinafter the same), saccharifying enzyme activity, and liquefying enzyme activity of *R. oryzae* M1 pure-culture Fuqu. All initially increased and then decreased. The maximum values of esterifying enzyme activity (74.66 U/g), saccharifying enzyme activity (415.36 U/g), and liquefying enzyme activity (0.465 U/g) were achieved at an incubation temperature of 30 °C. This indicated that below 30 °C, mycelial growth, spore germination, and hyphal expansion were slowed, resulting in insufficient biomass accumulation to produce the esterifying enzyme system and thus to lower esterifying enzyme activity. Above 30 °C, mycelial growth was inhibited, leading to a decline in overall metabolic capacity and a subsequent reduction in esterifying enzyme activity [24].

The effects of inoculation amount on the enzyme activities of *R. oryzae* M1 pure-culture Fuqu are shown in Figure 4B. As the inoculation amount increased, the esterifying enzyme activity, saccharifying enzyme activity, and liquefying enzyme activity of *R. oryzae* M1 pure-culture Fuqu initially increased and then decreased. The maximum values were achieved at an inoculation amount of 0.4%, reaching 75.84 U/g, 419.62 U/g, and 0.472 U/g, respectively. This indicated that at an inoculation amount of 0.4%, the microorganisms could fully utilize the resources to rapidly establish a dominant community and efficiently initiate both primary and secondary metabolism. When the inoculation amount was too low, the establishment of the microbial community was slow. Conversely, an overly high inoculation level resulted in either the early exhaustion of nutrients or the overproduction of inhibitory metabolites [25].

The effects of Huangshui addition amount on the enzyme activities of *R. oryzae* pure-culture Fuqu are shown in Figure 4C. As the Huangshui addition amount increased, the esterifying enzyme activity, saccharifying enzyme activity, and liquefying enzyme activity of *R. oryzae* M1 pure-culture Fuqu initially increased and then decreased. The maximum values were achieved at a Huangshui addition amount of 10%, reaching 78.61 U/g, 439.53 U/g, and 0.473 U/g, respectively. This phenomenon may be explained as follows: with the increase in the Huangshui addition amount, a slightly acidic environment was gradually established in the Fuqu. This condition was favorable for the growth of *R. oryzae* M1, thereby enhancing both biomass accumulation and metabolic activity [26]. However, when the Huangshui addition amount reached 20%, the esterifying enzyme activity was lower than that of the control group without Huangshui addition. This was likely because excessive addition resulted in an overly low environmental pH, which exceeded the optimal growth and metabolic range for the strain, ultimately leading to a decline in esterifying enzyme activity [27].

Based on the single-factor experiments, Huangshui addition amount (A), inoculation amount (B), and incubation temperature (C) were selected as influencing factors, with esterifying enzyme activity as the evaluation index, and a three-factor, three-level orthogonal experiment was conducted. The orthogonal experimental results are shown in Table 1. Analysis of the range (R) values indicated that the order of influence of each factor on esterifying enzyme activity was Huangshui addition amount (A) > inoculation amount (B) > incubation temperature (C). The optimal combination of culture conditions for *R. oryzae* pure-culture Fuqu was A_2_B_3_C_1_, namely, a Huangshui addition amount of 10%, an inoculation amount of 0.5%, and an incubation temperature of 30 °C. Under these optimized conditions, the esterifying enzyme activity of the pure-culture Fuqu prepared with *R. oryzae* M1 reached 80.13 U/g, the saccharifying enzyme activity reached 441.67 U/g, the liquefying enzyme activity reached 0.482 U/g, the spore count reached 1.73 × 10^9^ CFU/g, and the moisture content was 9.67%.

### 3.4. Optimization of Preparation Conditions for R. oryzae M1-Fortified Daqu

**Figure 5:**
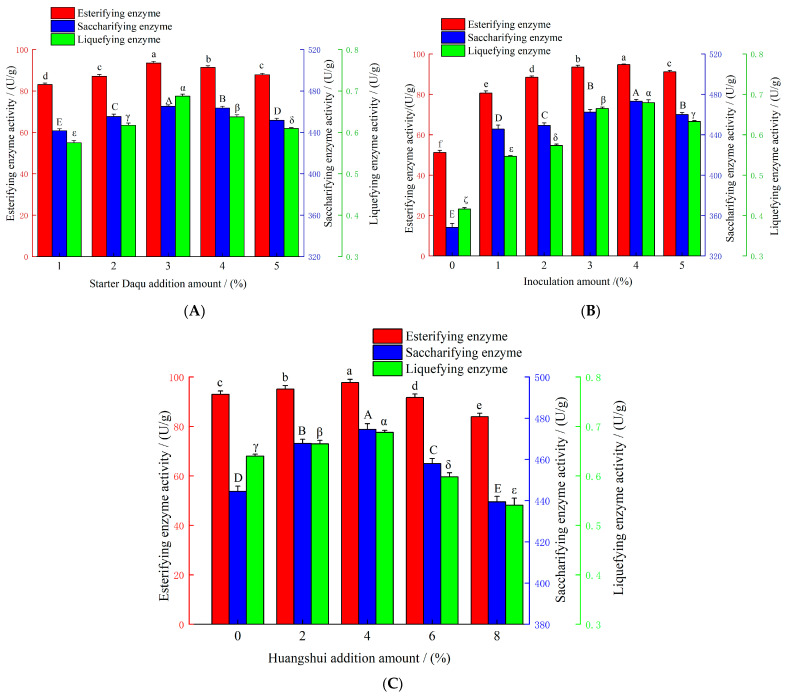
Effects of different factors on enzyme activities of *R. oryzae* M1-fortified Daqu. (**A**) Effect of starter Daqu addition amount on the esterifying, saccharifying, and liquefying enzyme activities of *R. oryzae* M1-fortified Daqu. (**B**) Effect of inoculation amount on the esterifying, saccharifying, and liquefying enzyme activities of *R. oryzae* M1-fortified Daqu. (**C**) Effect of Huangshui addition amount on the esterifying, saccharifying, and liquefying enzyme activities of *R. oryzae* M1-fortified Daqu. Different letters indicate significant differences at *p* < 0.05. The inoculation amount refers to *R. oryzae* Pure-culture Fuqu.

The effect of starter Daqu addition amount on the enzyme activities of *R. oryzae* M1-fortified Daqu is shown in Figure 5A. As the starter Daqu addition amount increased, the esterifying enzyme activity of fortified Daqu prepared with *R. oryzae* M1 initially increased and then decreased. When the starter Daqu addition amount was 3%, the esterifying enzyme activity, saccharifying enzyme activity, and liquefying enzyme activity of the fortified Daqu reached their maximum values of 93.75 U/g, 465.24 U/g, and 0.676 U/g, respectively, which were significantly higher than those obtained with other starter Daqu addition amounts. This may be explained as follows: a 3% starter Daqu addition amount represented a critical balance point. When the starter Daqu addition was too low, the acid-producing bacteria and other molds present in the starter Daqu could not provide sufficient growth factors, precursor substances, or a suitable environment for *R. oryzae* M1 [28]. Conversely, when the starter Daqu addition was too high, excessive competition for nutrients and space with *R. oryzae* M1 occurred, thereby inhibiting its enzyme production capacity and leading to a reduction in esterifying enzyme activity.

The effect of inoculation amount on the esterifying enzyme activity of *R. oryzae* M1-fortified Daqu is shown in Figure 5B. As the inoculation amount increased, the esterifying enzyme activity of fortified Daqu prepared with *R. oryzae* M1 initially increased and then decreased. When the inoculation amount ranged from 1% to 4%, the esterifying enzyme activity increased with increasing inoculation amount. This may be because a higher initial inoculation amount enabled the *R. oryzae* M1 population to more rapidly overcome the lag phase of adaptation and enter the exponential growth phase and the productive stationary phase earlier, thereby significantly enhancing the esterification efficiency per unit time [29]. At an inoculation amount of 4%, the esterifying enzyme activity, saccharifying enzyme activity, and liquefying enzyme activity of the fortified Daqu reached their maximum values of 94.74 U/g, 473.39 U/g, and 0.675 U/g, respectively. When the inoculation amount exceeded 4%, the esterifying enzyme activity showed a decreasing trend but remained significantly higher than that of the control group (0% inoculation). This could be attributed to excessive inoculation leading to rapid proliferation of the strain, swift depletion of nutrients, and accelerated aging of the mycelia, resulting in reduced esterifying enzyme production by the strain and, consequently, lower esterifying enzyme activity in the *R. oryzae* M1-fortified Daqu.

The effect of Huangshui addition amount on the esterifying enzyme activity of *R. oryzae* M1-fortified Daqu is shown in Figure 5C. As the Huangshui addition amount increased, the esterifying enzyme activity of fortified Daqu prepared with *R. oryzae* M1 initially increased and then decreased. The maximum values of esterifying enzyme activity (97.67 U/g), saccharifying enzyme activity (474.59 U/g), and liquefying enzyme activity (0.651 U/g) were achieved at a Huangshui addition amount of 4%. When the Huangshui addition amount ranged from 0% to 4%, a positive correlation was observed between esterifying enzyme activity and the Huangshui addition amount. This may be attributed to the fact that an appropriate amount of Huangshui promoted faster biomass accumulation and enhanced metabolic activity of the strain, thereby facilitating the synthesis and secretion of the esterifying enzyme system [30]. However, when the Huangshui addition amount exceeded 4%, a negative correlation was observed between the esterifying enzyme activity and the Huangshui addition amount. This decline could be explained by the excessive accumulation of acidic substances when the Huangshui addition surpassed a certain threshold, which may have significantly lowered the pH of the culture system beyond the optimal range for strain growth. Such an overly acidified environment may have not only inhibited normal mycelial growth and reproduction but also directly affected the activity and stability of the esterifying enzymes, ultimately leading to an overall reduction in esterification capacity [31].

Based on the results of single-factor experiments, Huangshui addition amount (A), inoculation amount (B), and starter Daqu addition amount (C) were selected as influencing factors, with esterifying enzyme activity as the evaluation index. A three-factor, three-level orthogonal experiment was conducted. The orthogonal experimental results and analysis are shown in Table 2. As shown in Table 2, analysis of the range (R) values indicated that the order of influence of each factor on esterifying enzyme activity was Huangshui addition amount (A) > inoculation amount (B) > starter Daqu addition amount (C). The optimal combination of conditions for fortified Daqu preparation was A_2_B_3_C_1_, namely a Huangshui addition amount of 4%, an inoculation amount of 5%, and a starter Daqu addition amount of 3%. Under these optimized conditions, the esterifying enzyme activity of the fortified Daqu prepared with *R. oryzae* M1 reached 103.22 U/g, the saccharifying enzyme activity reached 500.29 U/g, the liquefying enzyme activity reached 0.681 U/g, the moisture content was 12.09%, the total acidity was 1.03 mmol/10g, and the fermentation power was 0.41 U/g. All these physicochemical indices met the industry standards. Compared with the control group, the esterifying enzyme activity of the fortified Daqu was increased by 51.79%.

### 3.5. Significant Increase in the Relative Abundance of Rhizopus in Fortified Daqu

**Figure 6:**
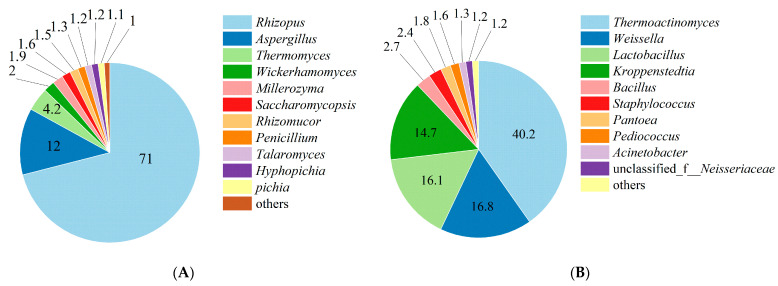
Microbial community structure at the genus level in *R. oryzae* M1-fortified Daqu. (**A**) Fungal community. (**B**) Bacterial community. Values are percentages.

Microbial community analysis of the fortified Daqu revealed that *Rhizopus* exhibited the highest abundance within the fungal community, with a relative abundance of 71% (Figure 6A). Compared with the non-fortified Daqu (Figure 1B), the proportion of *Rhizopus* was significantly increased, indicating that the fortification strategy was successfully implemented. These results suggest that the target functional strain (*R. oryzae* M1) may have achieved dominant colonization in the Daqu.

With respect to the fungal community, in addition to the absolute dominance of *Rhizopus*, *Aspergillus* (12%) was identified as the subdominant genus. A certain proportion of thermotolerant fungi (e.g., *Thermomyces*, 4.2%) was also detected, suggesting that the Daqu may have undergone a high-temperature fermentation phase. The overall abundance of yeasts was relatively low (each genus accounting for approximately 1–2%), though most were aroma-producing yeasts [32,33,34]. Regarding the bacterial community, the structure was dominated by the phylum Firmicutes (Figure 6B). *Thermoactinomyces* was the most abundant genus (40.2%), and the results suggest that the Daqu experienced a sufficiently high-temperature process. *Weissella* (16.8%) and *Lactobacillus* (16.1%) constituted the second largest group, representing important lactic acid bacteria. *Kroppenstedtia* (14.7%) was also identified as a dominant genus. Other functional bacteria, such as *Bacillus* (2.4%), along with several low-abundance taxa, were also detected [35,36,37].

In summary, the introduction of *R. oryzae* M1 successfully reshaped the microbial community structure of the fortified Daqu: *Rhizopus* became the absolute dominant fungus, while the bacterial community formed a synergistic ecological model dominated by functional bacterial groups such as *Thermoactinomyces* and lactic acid bacteria [38,39]. The impact of this altered microbial community structure on the fermentation process warrants further investigation [40,41].

### 3.6. Fortified Daqu Effectively Increases the Ester Content in Base Baijiu

**Figure 7:**
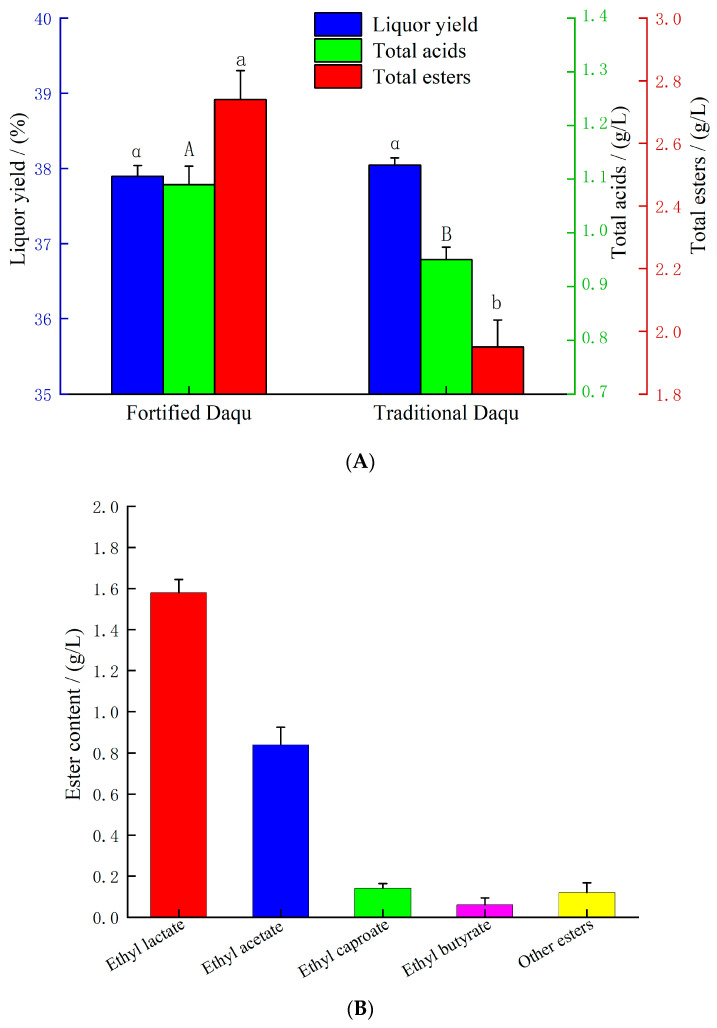
Yield and preliminary flavor analysis of base Baijiu. (**A**) total esters, total acids, liquor yield. Significant differences are indicated by different letters (*p* < 0.05). (**B**) Contents of various esters.

After 45 d of solid-state fermentation using the *R. oryzae* M1-fortified Daqu prepared under the optimal process conditions determined by the orthogonal experiment, the key indices of the base Baijiu are shown in Figure 7A. The total ester content of the base Baijiu in the experimental group was 2.74 g/L, which was significantly higher than that in the control group (1.95 g/L). The total ester content of the base Baijiu was 2.74 g/L. As shown in Figure 7B, the composition of the four major esters was as follows: ethyl lactate was the most abundant, with a concentration of 1.58 g/L, accounting for 54.0% of the total esters; ethyl acetate followed at 0.84 g/L, representing 30.7%; ethyl caproate and ethyl butyrate were present at lower levels of 0.14 g/L and 0.06 g/L, comprising 5.1% and 2.2%, respectively. Collectively, these four esters accounted for 2.62 g/L, or 95.6% of the total esters, with the remaining 0.12 g/L (4.4%) attributed to other esters (e.g., ethyl propionate and ethyl valerate). These results indicate that the ester profile was predominantly composed of these four compounds. Considering that the esterifying enzyme activity of the fortified Daqu in the experimental group was also significantly higher than that in the control group, it could be inferred that the difference in total ester content of the base Baijiu was mainly attributed to the improvement in the esterifying enzyme activity of the Daqu. Enhancing the esterifying enzyme activity of Daqu effectively promoted the esterification reaction between acids and alcohols in the fermentation system, thereby increasing the total ester content of the base Baijiu. Concurrently, the change in total acid content of the base Baijiu in the experimental group was closely related to its total ester level. Esters in the base Baijiu can undergo hydrolysis to generate the corresponding alcohols and organic acids; a higher ester content led to a greater production of acids through hydrolysis, ultimately manifesting as a correspondingly higher total acid content. Furthermore, no significant difference in the liquor yield was observed between the experimental group and the control group, with the overall levels being essentially consistent.

## 4. Conclusions

In this study, a technical system for producing functional fortified Daqu using *R. oryzae* M1 as the core strain was successfully established. The high esterifying enzyme-producing strain M1, screened from sub-high-temperature Daqu, was identified as *R. oryzae*. Through single-factor and orthogonal experiments, the optimal preparation conditions were determined for pure-culture Fuqu (10% Huangshui addition, 0.5% inoculation amount, 30 °C) and for *R. oryzae* M1-fortified Daqu (4% Huangshui addition, 5% inoculation amount, 3% starter Daqu addition). Under these optimized conditions, the esterifying enzyme activity of the fortified Daqu was increased by 51.79% compared with that of the control group. Microbial community structure analysis revealed that the relative abundance of the genus *Rhizopus* in the fortified Daqu was increased by 35% compared with that of the control, establishing it as the absolute dominant fungus and confirming the successful colonization of *R. oryzae* M1 and its pivotal role in enhancing esterifying enzyme activity. Application of this fortified Daqu in solid-state Baijiu fermentation yielded a base Baijiu with a total ester content of 2.74 g/L, representing a 40.5% increase compared with the control. This study systematically elucidates the preparation process of *R. oryzae* M1-fortified Daqu and its regulatory mechanism on microbial community structure and function, thereby providing a reproducible technical strategy and an excellent microbial strain for directionally enhancing Daqu quality and improving Baijiu flavor. These findings have significant implications for the modernization and advancement of traditional fermented foods.

## Figures and Tables

**Table 1:** Orthogonal experiment for esterifying enzyme activity of *R. oryzae* M1 pure-culture Fuqu.

Test No.	A	B	C	Activity/(U/g)
1	1 (5)	1 (0.3)	1 (30)	74.27
2	1	2 (0.4)	2 (33)	74.98
3	1	3 (0.5)	3 (36)	73.21
4	2 (10)	1	2	76.75
5	2	2	3	77.61
6	2	3	1	80.13
7	3 (15)	1	3	72.35
8	3	2	1	74.12
9	3	3	2	75.61
k1	74.15	74.46	76.17	
k2	78.16	75.57	75.78	
k3	74.03	76.32	74.39	
R	4.14	1.86	1.78	

Note: A, Huangshui addition amount (%); B, inoculation amount (%); C, incubation temperature/(°C). Activity refers to esterifying enzyme activity.

**Table 2:** Orthogonal experiment for esterifying enzyme activity of *R. oryzae* M1-fortified Daqu.

Test No.	A	B	C	Activity/(U/g)
1	1 (2)	1 (3)	1 (3)	95.47
2	1	2 (4)	2 (4)	96.41
3	1	3 (5)	3 (5)	96.97
4	2 (4)	1	2	99.34
5	2	2	3	101.84
6	2	3	1	103.22
7	3 (6)	1	3	93.61
8	3	2	1	94.12
9	3	3	2	93.53
k1	96.28	96.14	97.60	
k2	101.46	97.46	96.43	
k3	93.75	97.91	97.47	
R	7.70	1.77	1.17	

Note: A, Huangshui addition amount (%); B, inoculation amount (%); C, starter Daqu addition amount (%). Activity refers to esterifying enzyme activity.

## Data Availability

The original contributions presented in this study are included in the article. Further inquiries can be directed to the corresponding author.
